# Study on Shoulder Joint Parameters and Available Supraspinatus Outlet Area Using Three-Dimensional Computed Tomography Reconstruction

**DOI:** 10.3390/tomography10090100

**Published:** 2024-08-29

**Authors:** Xi Chen, Tangzhao Liang, Xiaopeng Yin, Chang Liu, Jianhua Ren, Shouwen Su, Shihai Jiang, Kun Wang

**Affiliations:** 1 Department of Joint and Trauma Surgery, Third Affiliated Hospital of Sun Yat-Sen University, Guangzhou 510630, China; chenx728@mail2.sysu.edu.cn (X.C.); liangtzh5@mail.sysu.edu.cn (T.L.); y839579487@163.com (X.Y.); liuch398@mail2.sysu.edu.cn (C.L.); renjianh@mail.sysu.edu.cn (J.R.); sushw3@mail.sysu.edu.cn (S.S.); 2 Orthopedics Surgery, Hanzhong People’s Hospital, Hanzhong 724200, China; 3 Institute of Laboratory Medicine, Clinical Chemistry and Molecular Diagnostics, University Hospital Leipzig, 04103 Leipzig, Germany

**Keywords:** computed tomography, 3D reconstruction, shoulder impingement, available supraspinatus outlet area, supraspinatus outlet area

## Abstract

Studies addressing the anatomical values of the supraspinatus outlet area (SOA) and the available supraspinatus outlet area (ASOA) are insufficient. This study focused on precisely measuring the SOA and ASOA values in a sample from the Chinese population using 3D CT (computed tomography) reconstruction. We analyzed CT imaging of 96 normal patients (59 males and 37 females) who underwent shoulder examinations in a hospital between 2011 and 2021. The SOA, ASOA, acromiohumeral distance (AHD), coracohumeral distance (CHD), coracoacromial arch radius (CAR), and humeral head radius (HHR) were estimated, and statistical correlation analyses were performed. There were significant sex differences observed in SOA (men: 957.62 ± 158.66 mm^2^; women: 735.87 ± 95.86 mm^2^) and ASOA (men: 661.35 ± 104.88 mm^2^; women: 511.49 ± 69.26 mm^2^), CHD (men: 11.22 ± 2.24 mm; women: 9.23 ± 1.35 mm), CAR (men: 37.18 ± 2.70 mm; women: 33.04 ± 3.15 mm), and HHR (men: 22.65 ± 1.44 mm; women: 20.53 ± 0.95 mm). Additionally, both SOA and ASOA showed positive and linear correlations with AHD, CHD, CAR, and HHR (R: 0.304–0.494, all *p* < 0.05). This study provides physiologic reference values of SOA and ASOA in the Chinese population, highlighting the sex differences and the correlations with shoulder anatomical parameters.

## 1. Introduction

Contemporary orthopedic practice and research requires that orthopedists possess a comprehensive understanding of the musculoskeletal anatomy [1,2,3]. Rotator cuff tear (RCT) is a common worldwide joint disorder that seriously affects shoulder joint function [4,5]. In 1972, Neer et al. [6] defined shoulder impingement as one of the causes of RCT, specifically implicating the involvement of the coracoacromial ligament. Subsequent research by Burns and Whipple [7] found that impingement occurred more frequently with the coracoacromial ligament than with the acromion. This led to the introduction of the concept of “supraspinatus outlet area (SOA)” and now a further recognition that “outlet impingement” significantly contributes to the development of RCT [8,9,10,11]. A deeper understanding of the diversity of SOA is necessary for guiding RCT therapy [12,13,14,15]. A previous study also described the concept of available supraspinatus outlet area (ASOA), indicating that the size of the area of the rotator cuff tear correlates with ASOA [10]. However, the general SOA or ASOA reference value from the normal population has not yet been studied.

Interestingly, gender differences in skeletal anatomy have been a reported risk factor in RCT re-tears [16]. Female gender was identified as an independent predictive factor for poor outcomes after RCT surgery [17]. One possible underlying mechanism is that women typically have a narrower acromiohumeral distance (AHD) and may be more prone to rotator cuff tears due to the inherently smaller ASOA. However, the association between AHD and ASOA remains largely unknown.

In addition, measurements of skeletal anatomy have evolved from simple 2D measurements to more precise three-dimensional (3D) measurements with computer modeling [1,18,19,20]. Advanced medical imaging techniques, such as 3D reconstruction technology, allow precise visualization of intricate anatomical structures, especially for assessments of the synovial joint space [21,22,23].

In this study, we utilized CT-based 3D reconstruction to measure SOA and ASOA. As the acromion and humeral head tend to be smaller in females, we hypothesized that females have a narrower SOA and ASOA compared to those of males. We further tested the correlation between ASOA and shoulder anatomical parameters (i.e., minimum acromiohumeral distance (AHD), coracohumeral distance (CHD), coracoacromial arch radius (CAR), and humeral head radius (HHR)). With this study, we aimed to report the reference values of SOA and ASOA in a sample from the normal population.

## 2. Materials and Methods

### 2.1. Patients

All procedures performed in studies involving human participants were in accordance with the ethical standards of the local institutional ethics committee and the 1975 Helsinki Declaration and its later amendments or comparable ethical standards. A total of 1713 patients underwent CT examinations of the shoulder at the Department of Radiology in a Chinese hospital between 2011 and 2021. The primary reason for patients undergoing CT scanning of the shoulder joint was suspicion of structural injuries in the upper limbs. Two senior orthopedic surgeons retrospectively reviewed the CT imaging and independently documented their diagnostic findings on the CT images. Any discrepancies in interpretation were resolved through consensus. After applying admission and exclusion criteria, a final analysis was conducted on a total of 96 cases (male, 59 cases; female, 37 cases).

### 2.2. Inclusion and Exclusion Criteria

**Figure 1:**
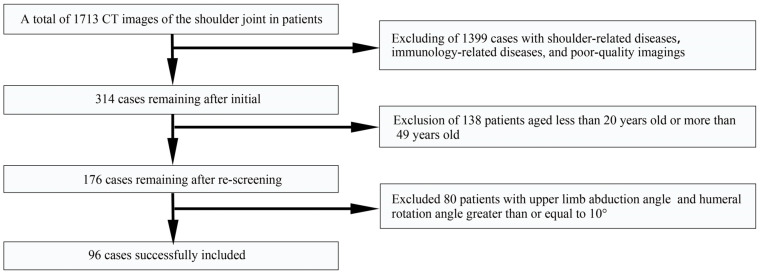
Flowchart diagram of the study. There were 1713 patients who received a standard shoulder CT examination, and ultimately, a total of 96 cases were included in the current study.

The inclusion criteria for this study were applied to all patients who had shoulder examinations conducted through the PACS system (Picture Archiving and Communication System) between 1 January 2011 and 31 December 2021. Patients who had an RCT, shoulder instability, shoulder fractures, shoulder osteoarthritis, scoliosis deformities, shoulder deformities, bone tumors of the upper limb, immunology-related diseases, and images with poor imaging quality were excluded. Additionally, patients younger than 20 years old or older than 49 years old were also excluded [24,25,26]. We further excluded patients with upper limb abduction angles (the angle between the axis of the proximal humerus and the line of the spinous process of the spine) and internal or external humeral rotation angles (the angle between the axis of the humeral head and the line of bone between the anterior and posterior edges of the glenoid cavity) less than 0° or greater than 10° (Figure 1) [27,28].

### 2.3. Scapular Coordinate System

**Figure 2:**
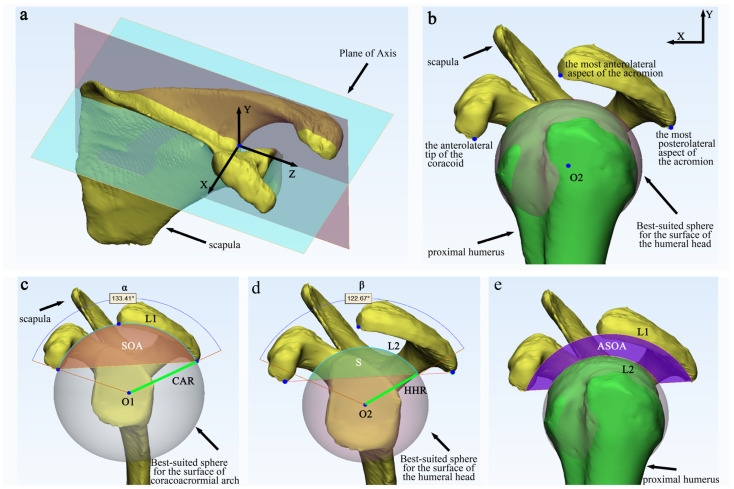
Creation of Coordinate Axes and Measurements of Associated Variables. The methodology for establishing axes and measurement variables is as follows: (**a**) A 3D bone surface model of the scapula serves as the basis for establishing a coordinate system. (**b**) The anterior and posterior reference points of the acromion, the coracoid reference point, and the sphere that best fits the surface of the humeral head are defined in the X-Y plane. (**c**) The supraspinatus outlet area is measured, along with other relevant values. (**d**) The size of the humeral head sphere that encroaches on the outlet area of the supraspinatus and the associated values are quantified. (**e**) The available area within the supraspinatus outlet is measured.

We established a coordinate system for the scapula based on previous studies [29,30]. Briefly, the medial-lateral (Z) axis is aligned with the scapular axis and is determined by connecting five points along the supraspinatus fossa projected onto the scapular plane. The coronal plane of the scapula is defined by the trigonum spinae, the inferior angle of the scapula, and the medial-most point of the five points that define the medial-lateral axis. The anteroposterior (X) axis is perpendicular to both the scapular plane and the *Z*-axis. The mediolateral (Y) axis is perpendicular to the other two axes. The origin of the coordinate system corresponds to the spinoglenoid notch, which aligns with the *Z*-axis of the scapula (Figure 2a).

### 2.4. Supraspinatus Outlet Area (SOA) and Available Supraspinatus Outlet Area (ASOA)

All data were obtained through semi-automatic measurements in the X-Y coordinate plane. Three crucial coordinate points were fixed: (1) the most posterolateral aspect of the acromion, (2) the most anterolateral aspect of the acromion corresponding to the most lateral aspect of the insertion of the coracoacromial ligament, and (3) the anterolateral tip of the coracoid corresponding to the insertion of the coracoacromial ligament (Figure 2b) [10]. Firstly, a circle is fitted using the most lateral aspect and anterior lateral aspect of the acromion and the anterior tip of the coracoid process to accurately represent the coracoacromial arch. The center of this circle is denoted as **O1,** and the radius is denoted as **CAR**. The arc formed by the most lateral aspect and anterior lateral aspect of the acromion and the anterior tip of the coracoid process is represented as **L1** and the angle as **α**. The area of the supraspinatus outlet is determined by the semicircle created by the anterior and posterior reference points of the acromion and the coracoid reference point, denoted as **SOA** (Figure 2c). Moving on, by enfolding the region from the most cranial to the most caudal of the humeral head, a sphere is created to best fit the surface of the humeral head. The center of this sphere is denoted as **O2,** and the radius is denoted as **HHR** (Figure 2d). The size of the area within the **SOA** that is encroached by the humeral head sphere is denoted as **S**, the arc length of the encroached area is denoted as **L2**, and the angle is denoted as **β** (Figure 2d). Finally, the available area within the supraspinatus outlet is calculated by subtracting S from SOA, and the result is recorded as **ASOA** (Figure 2e). To guarantee the precision and dependability of the measurements, a senior orthopedic surgeon performed three repetitions of the same anatomical parameter measurements and subsequently documented the mean value. To ensure the reproducibility of the data, one week later, the same investigator conducted a second set of measurements of the imaging data from 20 randomly selected patients (including 10 men and 10 women) and calculated the intraclass correlation coefficient of the observers.

**The following variables are defined and used as follows:**

**O1:** center of the best-suited sphere for the surface of the coracoacromial arch.

**CAR:** coracoacromial arch radius.

**L1:** length of the coracoacromial arch.

**A:** angle of the coracoacromial arc.

**SOA:** supraspinatus outlet area. 

**O2:** center of the best-suited sphere for the surface of the humeral head.

**HHR:** humeral head radius.

**L2:** arc length of the humeral head sphere within the supraspinatus outlet area.

**β:** angle of the arc length of the humeral head sphere within the supraspinatus outlet area. 

**S:** size of the area within the supraspinatus outlet area encroached by the humeral head sphere. 

**ASOA:** available area within the supraspinatus outlet.

### 2.5. The Minimum Acromiohumeral Distance and Coracohumeral Distance

We used previously established methods to measure the minimum AHD and CHD [28,31]. In brief, a 3D CT bone surface model of the scapula and proximal humerus were reconstructed using Mimics software (version 20.0; Materialise, Leuven, Belgium). The Meshlab software (version 2020.07; ISTI, Pisa, Italy) was employed to automatically measure the minimum AHD and CHD [28,31]. The distance between surfaces at any location could be evaluated through quantitative (computed Hausdorff Distances) and qualitative (obtained color-coded images) appraisals. The minimum AHD and CHD distances represent the minimum distance required to reach any point on the humeral head surface model from either the acromion surface model or the coracoid process surface model.

### 2.6. Data Process and Statistical Analysis

SPSS Statistics 27.0.1 (IBM Corporation, Armonk, NY, USA) was used for statistical analyses. The data for men and women were normally distributed using the Shapiro–Wilk test (*p* > 0.05). The independent samples test was used to assess sex differences in the measured parameters, and *p* values, 95% confidence intervals, and effect sizes were recorded. Interobserver reliability was assessed using the intraclass correlation coefficient (ICC), with a 95% confidence interval. Pearson’s correlation coefficient and linear regression analysis were used to assess the correlation between the parameters (SOA, ASOA, AHD, CHD, CAR, and HHR). Data measurements were presented uniformly using the mean ± standard deviation. Statistical significance was determined at *p* < 0.05.

## 3. Results

In this study, we analyzed images of the normal shoulder joints in 59 males (61.5%) and 37 females (38.5%). The final analysis included 44 left shoulders (45.8%) and 52 right shoulders (54.2%). The mean age of the study sample was 34.17 ± 8.58 [age range: 20–49 years]. The mean abduction angle of the upper limb in the study sample was 4.04 ± 3.20°, the mean internal rotation was 6.07 ± 2.80°, and the mean external rotation was 5.77 ± 3.13° (Table 1).

### 3.1. Supraspinatus Outlet Area and Available Outlet Area

**Figure 3:**
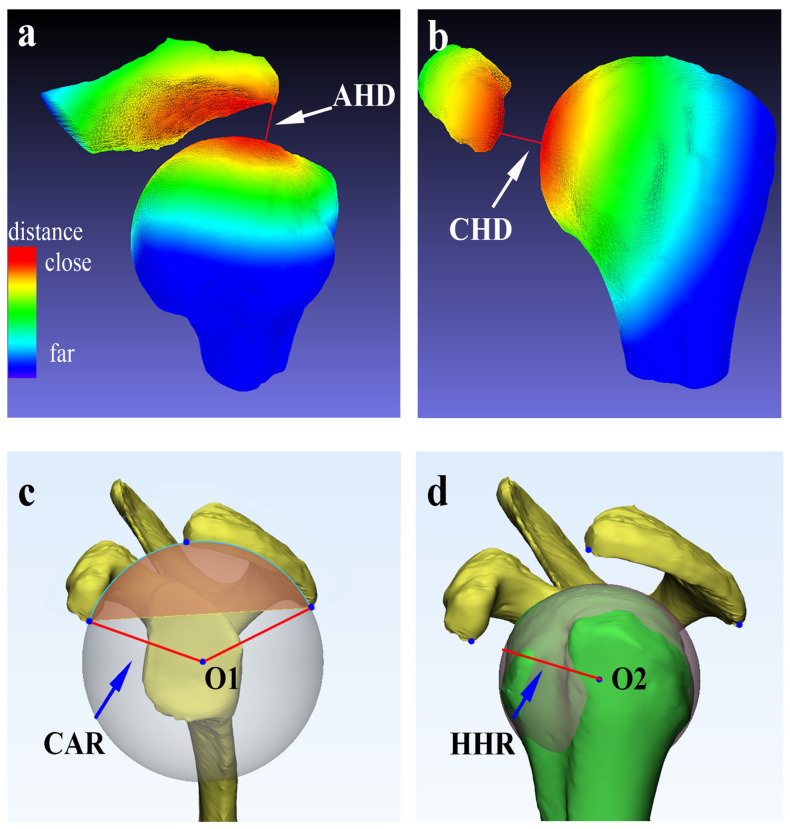
Representative demonstration of AHD, CHD, CAR, and HHR parameters in the reconstructed 3D models. The red areas indicate the minimum distances from (**a**) the acromion and (**b**) coracoid process to the humeral head. The sequential color change from red to yellow, green, and blue represents a gradual increase in the distance between the two surface models. Panel (**c**) presents the radius of the best-suited sphere for the surface of the coracoacromial arch. Panel (**d**) presents the radius of the best-suited sphere for the surface of the humeral head. AHD, acromiohumeral distance; CHD, coracohumeral distance; CAR, coracoacromial arch radius; HHR, humeral head radius; O1, center of the best-suited sphere for the surface of the coracoacromial arch; O2, center of the best-suited sphere for the surface of the humeral head.

We first measured the minimum AHD (acromiohumeral distance) and CHD (coracohumeral distance) using previously established methods to show the reliability of the current methods and the sex differences comparisons. As shown in Figure 3a,b, the red highlighted areas indicate the minimum distances from the acromion and coracoid process to the humeral head. Sex differences were identified for CHD (females, 9.23 ± 1.35 mm, *p* < 0.001, 95% CI = [1.26, 2.72], d = 1.02), while no significant differences were observed for AHD, indicating the minimum AHD is comparable between healthy females and males (age younger than 49 years old).

We further assessed the CAR (coracoacromial arch radius) and HHR (humeral head radius) for the determination of the ASOA/SOA ratio (Figure 3c,d). Sex differences were identified for SOA, S, ASOA, CAR, HHR, L1, and L2, while no differences were observed for α, β, HHR/CAR, and the ASOA/SOA ratio. Among males, the values of SOA (957.62 ± 158.66 mm^2^), ASOA (661.35 ± 104.88 mm^2^), CAR (37.18 ± 2.70 mm), and HHR (22.65 ± 1.44 mm) were significantly greater than those in females (SOA: 735.87 ± 95.86 mm^2^, t (94) = 7.66, *p* < 0.001, 95% CI = [170.17, 273.34], d = 1.61; ASOA: 511.49 ± 69.26 mm^2^, t (94) = 7.70, *p* < 0.001, 95% CI = [114.56, 185.16], d = 1.61; CAR: 33.04 ± 3.15 mm, t (94) = 6.84, *p* < 0.001, 95% CI = [2.93, 5.33], d = 1.43; HHR: 20.53 ± 0.95 mm, t (94) = 7.94, *p* < 0.001, 95% CI = [1.63, 2.60], d = 1.67). 

Interestingly, there were no sex differences observed for HHR/CAR (men: 0.61 ± 0.06, women: 0.63 ± 0.05, *p* = 0.364) and the ASOA/SOA ratio (men: 0.69 ± 0.03, women: 0.70 ± 0.03, *p* = 0.627). Of note, intra-observer reliability was excellent, with 95% ICCs ranging from 0.942 to 0.978 (Table 2).

### 3.2. Correlations between Shoulder Anatomical Parameters and SOA and ASOA

SOA exhibited a positive linear correlation with AHD (452.825 + 54.830 × AHD, R = 0.310, *p* = 0.02), CHD (580.784 + 27.886 × CHD, R = 0.345, *p* = 0.001), CAR (333.359 + 15.142 × CAR, R = 0.304, *p* = 0.003), and HHR (52.939 × HHR − 283.720, R = 0.494, *p* < 0.001). 

**Figure 4:**
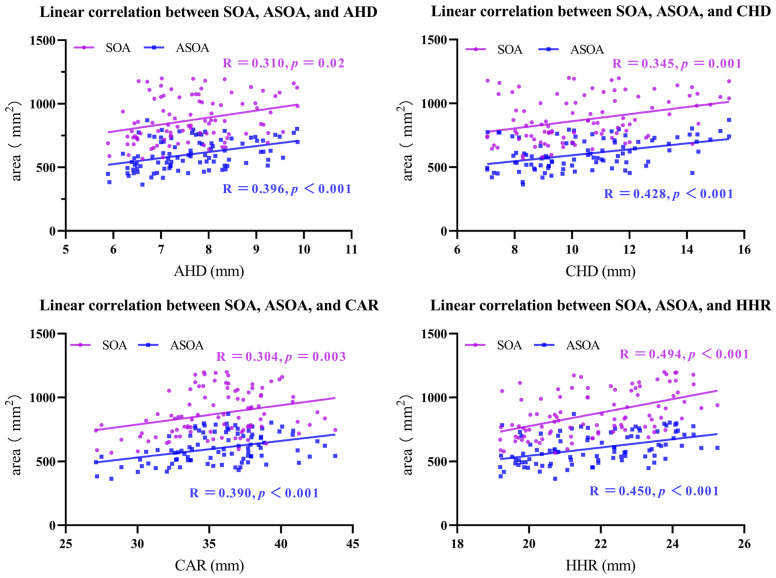
Linear regression relationship between AHD, CHD, CAR, or HHR and SOA and ASOA. SOA increases with increases in AHD, CHD, CAR, or HHR. SOA, supraspinatus outlet area; ASOA, available area within the supraspinatus outlet; AHD, acromiohumeral distance; CHD, coracohumeral distance; CAR, coracoacromial arch radius; HHR, humeral head radius.

Interestingly, ASOA also exhibited a positive linear correlation with AHD (241.824 + 47.304 × AHD, R = 0.396, *p* < 0.001), CHD (360.051 + 23.308 × CHD, R = 0.428, *p* < 0.001), CAR (137.721 + 13.092 × CAR, R = 0.390, *p* < 0.001), and HHR (32.461 × HHR − 105.166, R = 0.450, *p* < 0.001). These findings revealed that increases in AHD, CHD, CAR, or HHR corresponded to gradual increases in SOA and ASOA (Figure 4).

## 4. Discussion

The anatomical relationship between the coracoacromial arch and the humeral head is a crucial factor in the development of RCT. While previous studies have extensively investigated the association of shoulder joint anatomy with RCT, there is limited research on the spatial relationships between the coracoacromial arch and the humeral head [12,13,14,15]. Understanding these spatial relationships is important, as impingement syndrome suggests a “space” issue. In this study, we utilized 3D CT reconstruction to precisely measure SOA and ASOA in the Chinese population, aiming to enhance our understanding of shoulder joint anatomy. 

Our results indicated significant differences between males and females for several parameters, including SOA, ASOA, CAR, and HHR. Males exhibited larger values for these parameters compared to females, indicating potential anatomical gender differences. These differences may be attributed to variations in shoulder joint anatomy, thereby contributing to the prevalence of RCT development. Additionally, increases in AHD, CHD, CAR, or HHR values correlated with higher SOA and ASOA values, suggesting that outlet impingement may represent a “size mismatch” between the components of the supraspinatus outlet, i.e., the coracoacromial arch and the humeral head.

Previous studies have reported that the mean HHR value for the Chinese population is 22.1 ± 1.9 mm, which is similar to our findings (21.83 ± 1.63 mm) but smaller than that of Western populations (23.6 ± 2.4 mm) [1]. Interestingly, Gohlke et al. [14] found that the mean CAR was larger than the mean HHR by 39% in German individuals, which is very similar to our findings for HHR/CAR (0.62 ± 0.06). These results indicate that there already exists a regular “size match” between the coracoacromial arch and the humeral head in the normal population.

In comparison to a previous study by Zuckerman et al. [10], the SOA and ASOA values obtained in this study were larger. Zuckerman et al. [10] measured the SOA and ASOA of 140 shoulders from 70 cadaveric 3D models of the shoulder joint and reported values of 656.8 mm^2^ and 433.6 mm^2^, respectively. These values are significantly smaller than the measurements obtained in our study (872.15 ± 175.00 mm^2^ and 603.59 ± 117.94 mm^2^). We believe that this difference primarily arises from variances in the measurement methods used. We employed the same landmarks as Zuckerman et al. [10]. However, they defined ASOA as a triangular region below the coracoid process, whereas we defined it as a semicircular area. Considering the curved undersurface of the acromion and the elasticity of the coracoacromial ligament, we propose that the actual ASOA is a semi-oval area that conforms to the surface of the acromion below the coracoacromial ligament [32,33,34]. Age may also be a significant confounding factor for SOA and ASOA values. Previous studies suggest that age-related changes in bony spurs and the coracoacromial ligament can lead to narrowing of the SOA and ASOA [25,32,35,36,37]. To narrow this potential bias, we excluded patients older than 49 years in our study, emphasizing intrinsic anatomical factors that predispose individuals to outlet impingement.

The current study employs a few complex anatomical measurement parameters. However, the parameters were selected based on the findings of previous studies. Previous investigations indicated that the narrowing of the AHD and CHD correlated with the development of rotator cuff disease. In addition, a smaller coracoacromial arc angle may act as a predisposing factor of RCT. Additionally, the disappearance of the ‘size match’ between the CAR and HHR may be an indicator of RCT. To some extent, our study provided a comprehensive description of the anatomy of the shoulder joint by measuring multiple parameters. It is these detailed data that allow us to accurately characterize the features and the associations between SOA and ASOA and the anatomical parameters. Our results may have important clinical implications for understanding the development of RCT, especially regarding the influence of sex differences in the development of RCT. SOA and ASOA measurements provide quantitative data that can aid in the assessment of shoulder joint pathology in future studies.

This study has several limitations. Firstly, the study has a limited sample size. To enhance the reliability of the results for establishing reference values in a sample from a normal population, a strict screening process was undertaken. This process excluded any cases with factors that could cause abnormalities in the skeletal structure of the shoulder joint, so the final total was 96 cases. More participants would likely cause a reduction in bias during the selection process. Secondly, the sample size of the RCT group was lacking in the current study, leading to fewer clinical values for RCT detection. However, as a proof-of-concept investigation, we intend to design and establish the physiological reference values of SOA and ASOA in the normal population, thereby providing fundamental anatomical data of the shoulder joint. One of the innovations in our study is that only healthy people were studied. To the best of our knowledge, there are no clinical studies with a large number of participants evaluating shoulder SOA and ASOA parameters in normal populations, especially using accurate 3D reconstruction measurements. Last but not least, the retrospective design of this study and the reliance on CT data may introduce selection bias. Future studies should include a larger and more diverse population, utilize more comprehensive imaging techniques such as magnetic resonance imaging (MRI) for 3D validation, and further investigate the relationships between shoulder joint anatomy and RCT.

## 5. Conclusions

In conclusion, this study provides physiologic reference values for SOA and ASOA in a sample from the Chinese population, highlighting the sex differences and the correlations with shoulder anatomical parameters (AHD, CHD, CAR, and HHR). Our findings led to a better understanding of shoulder anatomical parameter values from a healthy population.

## Figures and Tables

**Table 1:** Participant characteristics.

Characteristic	All	Male	Female	*p* Value
Age, years	Age, years	31.95 ± 7.88 (*n* = 59)	37.70 ± 8.57 (*n* = 37)	0.001 *
Left shoulder, cases	44	26	18	
Right shoulder, cases	52	33	19	
Abduction angles, °	4.04 ± 3.20 (*n* = 96)	3.90 ± 3.16 (*n* = 59)	4.26 ± 3.29 (*n* = 37)	0.595
Internal rotation angles, °	6.07 ± 2.80 (*n* = 50)	5.98 ± 2.92 (*n* = 32)	6.23 ± 2.65 (*n* = 18)	0.760
External rotation angles, °	5.77 ± 3.13 (*n* = 46)	6.07 ± 3.01 (*n* = 27)	5.34 ± 3.32 (*n* = 19)	0.441

The data are presented as mean ± standard deviation, and the number of cases is enclosed in parentheses. * *p* value < 0.05 was considered statistically significant after testing.

**Table 2:** Sex differences in geometric measurements.

Variable	Male	Female	*p* Value	95% CI	Cohen d	95% ICC
AHD, mm	7.80 ± 1.02	7.40 ± 0.89	0.054	−0.01, 0.80		
CHD, mm	11.22 ± 2.24	9.23 ± 1.35	<0.001 *	1.26, 2.72	1.02	
CAR, mm	37.18 ± 2.70	33.04 ± 3.15	<0.001 *	2.93, 5.33	1.43	0.949
HHR, mm	22.65 ± 1.44	20.53 ± 0.95	<0.001 *	1.63, 2.60	1.67	0.944
HHR/CAR	0.61 ± 0.06	0.63 ± 0.05	0.292	−0.04, 0.01		
L1, mm	81.53 ± 5.43	71.70 ± 4.21	<0.001 *	7.87, 11.80	1.97	0.977
L2, mm	46.17 ± 4.11	40.46 ± 2.69	<0.001 *	4.33, 7.09	1.57	0.970
α, (°)	126.31 ± 12.18	125.08 ± 10.23	0.612	−3.55, 6.00		0.942
β, (°)	116.49 ± 10.56	114.65 ± 8.25	0.341	−1.99, 5.68		0.977
SOA, mm^2^	957.62 ± 158.66	735.87 ± 95.86	<0.001 *	170.17, 273.34	1.61	0.971
S, mm^2^	296.27 ± 65.51	224.38 ± 37.50	<0.001 *	51.00, 92.79	1.27	0.978
ASOA, mm^2^	661.35 ± 104.88	511.49 ± 69.26	<0.001 *	114.56, 185.16	1.61	0.965
ASOA/SOA	0.69 ± 0.03	0.70 ± 0.03	0.652	−0.02, 0.01		

The data are presented as mean ± standard deviation, with the range in parentheses. AHD, acromiohumeral distance; CHD, coracohumeral distance; CAR, coracoacromial arch radius; HHR, humeral head radius; L1, length of the coracoacromial arch; L2, arc length of the humeral head sphere within the supraspinatus outlet area; α, angle of the coracoacromial arc; β, angle of arc length of the humeral head sphere within the supraspinatus outlet area; SOA, supraspinatus outlet area; S, size of the area within supraspinatus outlet area encroached by the humeral head sphere; ASOA, available area within the supraspinatus outlet. * *p* value < 0.05 was considered statistically significant after testing.

## Data Availability

The datasets used and/or analyzed during the current study are available from the corresponding author on reasonable request.
